# A companion to the preclinical common data elements and case report forms for neuropathology studies in epilepsy research. A report of the TASK3 WG2 Neuropathology Working Group of the ILAE/AES Joint Translational Task Force

**DOI:** 10.1002/epi4.12638

**Published:** 2022-09-22

**Authors:** Eleonora Aronica, Devin K. Binder, Meinrad Drexel, Chrysanthy Ikonomidou, Shilpa D. Kadam, Guenther Sperk, Christian Steinhäuser

**Affiliations:** ^1^ Amsterdam UMC, University of Amsterdam, Department of (Neuro) Pathology Amsterdam Neuroscience Amsterdam The Netherlands; ^2^ Stichting Epilepsie Instellingen Nederland (SEIN) Heemstede The Netherlands; ^3^ Center for Glial‐Neuronal Interactions, Division of Biomedical Sciences, School of Medicine University of California Riverside California USA; ^4^ Department of Genetics and Pharmacology, Institute of Molecular and Cellular Pharmacology Medical University Innsbruck Innsbruck Austria; ^5^ Department of Neurology University of Wisconsin Madison Wisconsin USA; ^6^ The Hugo Moser Research Institute at Kennedy Krieger Baltimore Maryland USA; ^7^ Department of Neurology Johns Hopkins University School of Medicine Baltimore Maryland USA; ^8^ Department of Pharmacology Medical University Innsbruck Innsbruck Austria; ^9^ Institute of Cellular Neurosciences, Medical School University of Bonn Bonn Germany

**Keywords:** common data elements, epilepsy, inflammation model, neuropathology, preclinical, rodent

## Abstract

The International League Against Epilepsy/American Epilepsy Society (ILAE/AES) Joint Translational Task Force initiated the TASK3 working group to create common data elements (CDEs) for various aspects of preclinical epilepsy research studies, which could help improve the standardization of experimental designs. This article addresses neuropathological changes associated with seizures and epilepsy in rodent models of epilepsy. We discuss CDEs for histopathological parameters for neurodegeneration, changes in astrocyte morphology and function, mechanisms of inflammation, and changes in the blood‐brain barrier and myelin/oligodendrocytes resulting from recurrent seizures in rats and mice. We provide detailed CDE tables and case report forms (CRFs), and with this companion manuscript, we discuss the rationale and methodological aspects of individual neuropathological examinations. The CDEs, CRFs, and companion paper are available to all researchers, and their use will benefit the harmonization and comparability of translational preclinical epilepsy research. The ultimate hope is to facilitate the development of rational therapy concepts for treating epilepsies, seizures, and comorbidities and the development of biomarkers assessing the pathological state of the disease.

**Abbreviations**
ACD: accidental cell death
Apaf‐1: apoptotic protease activating factor 1, apoptosis marker
AQP4: aquaporin‐4, water channel
BBB: blood‐brain barrier
BrdU: bromo‐deoxy‐uridine
Breg cell: regulatory B cells
CaMK II: Ca^2+^/calmodulin‐activated protein kinase II
CASP: caspase, apoptosis marker
CCK: cholecystokinin‐octapeptide, marker of neuronal subtype
CD11b: cluster of differentiation 11b, marker of microglia activation
CD127: interleukin‐7 receptor‐α, subunit
CD13: pericyte marker
CD163: monocyte/macrophage marker
CD20: B‐lymphocyte antigen CD20
CD25: alpha‐chain of the IL‐2‐receptor, T‐cell marker
CD3, CD4: cluster of differentiation 3, 4, T‐cell marker
CD31: platelet/endothelial cell adhesion molecule‐1, marker of endothelial surface protein
CD68: cluster of differentiation 68, activation marker, microglia activation
CDE: common data element
*cfos, ΔFosB and Arc*
: transcription factors, marker for neuronal activity
CNPT: 2′,3′‐cyclic‐nucleotide 3′‐phosphodiesterase, myelin marker
CRF: case report form
CX3CR1: CX3C chemokine receptor 1, fractalkine receptor, activated microglia, and other myeloid cells
CyTOF: cytometry by time‐of‐flight mass spectrometry
DCX: doublecortin, marker for newly born cells
EAAT1, 2, 3, 5: excitatory amino acid transporters, astrocyte markers
foxP3: transcription factor, marker of subtype of CD4+ T‐cells (tregs)
GAD: glutamate decarboxylase, marker for GABA interneurons
GFAP: glial fibrillary acidic protein, astrocyte marker
Iba‐1: ionized binding adaptor molecule‐1, microglia activation marker
IHC: immunohistochemistry
IL‐1: interleukin‐1
IL‐10: marker for breg (B10) cells
ISH: in situ hybridization
KA: kainic acid
Ki‐67: proliferation marker, marker for newly born cells
LC3, LCB3: marker of autophagosomes
LFB stain: luxol fast blue stain, for oligodendrocytes
LOX: lipoxygenase
MAP‐2: microtubule associate protein 2, dendritic marker
MBP: myelin basic protein, myelin marker
MOG: myelin oligodendrocyte glycoprotein, myelin marker
MPT: mitochondrial permeability transition, cell death mechanism
NCDD: Nomenclature Committee on Cell Death
NeuN: neuronal nuclear antigen, neuronal marker
NF‐H subunit: neurofilament heavy subunit, marker, in soma and dendrites
NKB: neurokinin B, marker of neuronal subtype
NOS: nitric oxide synthetase, marker of neuronal subtype
NPY: neuropeptide Y, marker of neuronal subtype
olig2: oligodendrocyte lineage transcription factor 2
p‐MLKL: phosphorylated mixed lineage kinase domain‐like protein, necroptosis marker
P2X4, P2Y6, P2X7, P2Y12: purinergic receptors
PCNA: proliferating cell nuclear antigen, marker for newly born cells
PDGFRb: platelet‐derived growth factor receptor‐β, marker for pericytes
RCD: regulated cell death
RECA‐1 antibody: detecting a surface protein of endothelial cells
RIPK1 and RIPK3: receptor‐interacting serine/threonine‐protein kinases 1 and 3, markers of necroptosis
ROS: reactive oxygen species
SE: status epilepticus
TBI: traumatic brain injury
TGF‐β: transforming growth factor beta, breg cell (B10 cell) marker
Tie2: tyrosine kinase 2, the receptor for angiopoetin 1, marker for surface protein of endothelial cells
TLE: temporal lobe epilepsy
Tmem119: transmembrane protein 119, microglia‐specific marker
Terg cells: regulatory CD4+ T‐cells
VEGF: vascular epithelial growth factor, astrocyte marker
VGAT: vesicular GABA transporter, marker of GABA neurons (axons)
VGLUT1 and 2: vesicular glutamate transporter‐1 and 2, marker for glutamate neurons
VIP: vasoactive intestinal polypeptide, marker of neuronal subtype; The working group was coordinated by Guenther Sperk (Department of Pharmacology, Medical University Innsbruck, Innsbruck, Austria, guenther.sperk@i‐med.ac.at) and Eleonora Aronica (Amsterdam UMC, University of Amsterdam, Department of (Neuro)Pathology, Amsterdam Neuroscience, Amsterdam, The Netherlands, e.aronica@amsterdamumc.nl).

**Key Points**

- This joint ILAE/AES initiative introduces common data elements related to the assessment of various neuropathological parameters in adult rodents.
- Case report forms and a companion paper discussing the rationale and methods for neuropathological examinations are provided.
- Future use of these forms may help to harmonize animal experiments and to improve and facilitate data sharing and metaanalysis studies.

## 1 INTRODUCTION

This article represents a companion to common data elements (CDEs) and case report forms (CRFs) for evaluation of neuropathology in rodent (mouse and rat) models used in epilepsy research, and aims to facilitate understanding of the importance of assessing the neuropathological status in preclinical epilepsy research. It is part of the ongoing initiative by the TASK3 group of the International League Against Epilepsy/American Epilepsy Society (ILAE/AES) to CDEs for epilepsy research studies for optimizing the study design, data reporting, and interpretation across studies.^1^ The current working groups are aiming in establishing CDEs for studies on genetic and pediatric animal models, neuropathology, general pharmacology, imaging, and phenotyping in preclinical epilepsy research. Previous reports^2^ have established CDEs for preclinical studies on neurobehavioural comorbidities,^3^ pharmacology,^4^ physiology,^5^ EEG,^6^ and data management.^7^

When assessed in animal models or in the *postmortem* human brain, neuropathological changes such as neurodegeneration, gliosis, or inflammation have been found to commonly result from the underlying pathological processes and epileptic seizures. These may, however, also influence the clinical characteristics of the epilepsy phenotype (e.g., frequency and severity of seizures) or contribute to its establishment. In humans, *postmortem* tissue or tissue obtained at the surgery during resection of an epileptic focus (hippocampus, amygdala/hippocampus, and dysplastic cortex) is most commonly used for neuropathological assessment. In contrast, animal experiments allow for the study of the development of neuropathological changes and their underlying mechanisms; for example, by setting time points of investigation or by influencing the manifestation of individual neuropathological changes, for example, by pharmacological intervention or using transgenic animals. Thus, it is possible to also investigate the impact of specific neuropathological changes on the development of single seizures or epilepsy. Another important aspect is the impact of early life seizures on postnatal brain development, which also can be investigated in experimental animals.

In the CDEs and in this companion paper, we present a broad overview of neuropathological examinations helping to characterize animal epilepsy or seizure models and to give a detailed background to the neurological status of the animals in addition to their behavior and/or EEG. Such models have demonstrated usefulness in studies aiming to evaluate the effectiveness of treatments for seizures or epilepsies, and their impact on the epileptogenic process. The procedures described here are for rodent (mouse and rat) tissue, as mice and rats are the most common species used in epilepsy research. It is important to emphasize that neuropathological changes such as acute neurodegeneration or inflammation are not seen equally in all epilepsy models. While such features are especially prominent in models based on an initial episode of status epilepticus (SE) or head trauma, they may be minimal or absent in, for example, some genetic models of epilepsy or in kindling.^8^ In reverse, animal models may be designed to recapitulate certain neuropathological features (e.g., specific degeneration of certain neurons) to study their influence on seizures and epilepsy. Thus, neuropathological examinations are crucial for the characterization of the individual models and for investigating pathophysiological processes underlying the model. In most instances, only a subset of the neuropathological aspects listed in this companion paper and the respective CDEs will be investigated depending on the individual animal model to be characterized and/or the specific research goal to be addressed. For instance, in post‐SE models characterization of the extent and area of neurodegeneration and activation of astrocytes and microglia may be most essential and can be accompanied by the assessment of more detailed neuropathological aspects.

The CDEs presented here apply to adult rodents, rats, or mice, and are not readily applicable to immature animals, due to the variable postnatal vulnerability to hypoxia and sensitivity to compounds inducing epilepsy^9^, ^10^ in part caused by the dynamic postnatal development of glutamate and GABA receptors.^11^

We are dividing the CRFs, CDEs, and the companion paper into the following sections:

1. Basic parameters: animals and their health status.
2. Euthanasia, preparation, and collection of the tissue.
3. Neurodegeneration: pathology associated with neurons and neuronal pathways.
4. Pathology related to astrocytes.
5. Epilepsy‐induced immune responses.
6. Oligodendrocytes, myelin, and blood‐brain barrier (BBB).

In this accompanying paper, we aim to provide brief introductions on the background of each neuropathological test suggested. Most tests are based on basic immunohistochemistry (IHC) or in situ hybridization (ISH) procedures. Other tests, however, like Nissl stain, FluoroJade B, or the bromo‐deoxy‐uridine (BrdU) method require individual procedures. We are providing references and concise background on these methods. It is obvious that not all procedures are always required for the neuropathological characterization of an animal model or animal treatment. It is up to the researcher to set the individually required focus for the respective study.

## 2 METHODS

The forms are constructed analogously to previous preclinical CDEs by the TASK3 group of the ILAE/AES Joint Translational Task Force,^1^, ^2^, ^3^, ^4^, ^5^, ^6^ and complement efforts from other groups that generated similar CDEs.^12^ The proposed recommendations are based on previous work on neuropathology in animal models, notably models initiating epilepsy through provoking an episode of SE. Based on a critical review of literature, expert surveys, and discussions within the TASK3 Working Group 2 (WG2) Neuropathology Working Group of the ILAE/AES Joint Translational Task, we present consensus proposals reached by consensus of our working group members on neuropathological examination in animal models of epilepsy, including specific suggestions on histological stains and immunohistochemical markers to evaluate the major neuropathological features addressed in the specific sections and related CDEs. Evaluation of consensus between working group members was tested and refined by a Delphi process^13^: We applied a two‐round consensus process. In the first round, working group members voted blind to the other working group members on the selection and usefulness of the proposed neuropathological measures listed in the preliminary CRFs either by “agreeing,” “undecided,” or “disagreeing.” Participants were also asked to suggest any additional potential parameters or possible modifications. The votes were collected by E‐mail and included in an Excel sheet. After this first round of voting, the recommendations with undecided and disagreeing votes were discussed once more and revised according to the provided reasons for disagreement. The revised statements were voted on again and the results were again transferred to the Excel sheet and evaluated.

### 2.1 Pitfalls and troubleshooting for IHC and ISH

IHC and ISH are commonly used methods for detecting protein or RNA (mRNA, noncoding RNA) expression. They are reliable and attractive methods that can be applied to both frozen and formalin‐fixed, paraffin‐embedded (FFPE) tissues. IHC and ISH require standardized procedures and appropriate selection of antibodies and probes.

The most frequently used methods for neuropathological examinations are IHC/immunofluorescence and ISH. It would go far beyond the aim of this article to explain possible procedures in detail or even to provide protocols. These can be found in the extensive existing literature. For basic explanations, we include some links to papers/protocols by commercial suppliers of antibodies in an Appendix at the end of the manuscript (prior to the reference list). For IHC it is important to choose an appropriate staining method (often horse radish peroxidase). Often the immunohistochemical signal may be amplified by the use of appropriate strategies (e.g., peroxidase‐antiperoxidase method, streptavidin‐biotin‐peroxidase system, use of secondary antibodies, and use of amplification procedures like tyramide signal amplification^14^, ^15^). Immune fluorescence is mostly more sensitive than regular IHC and it allows double‐ and triple‐labeling of different antigens on the same section.

Similarly, for ISH also the appropriate method has to be chosen and adapted in the laboratory. There are two different approaches, the use of radiolabeled probes or nonradioactive (mostly using fluorescence labeled probes) procedures. Radiolabeled probes have the advantage of allowing quantitative evaluation, whereas it may be more practical to work with fluorescence‐labeled probes (fluorescence in situ hybridization^16^). In Tables 1 and 2, we are including key points regarding the performance of immunohistochemical procedures and ISH, respectively. There is some further advice on the methodologies in the Appendix at the end of the paper.

**TABLE 1:** Key points regarding the identification of cellular components to be considered using immunohistochemistry

Use the appropriate brain tissue sample preparation for each specific antibody (i.e., frozen and/or formalin fixed paraffin‐embedded (FFPE) tissue). Fixation, tissue processing, and target retrieval methods are important elements.^17^ Identify a minimum panel to characterize different cellular components. Read carefully the antibody datasheets including the list of the applications that have been successfully tested with the specific antibody. Consider carefully cross‐reactivity between antigens (e.g., two antigens with similar structural regions that the antibody can recognize). Consider carefully the different cross‐reactivity with the homologous proteins in nonhuman models, such as mouse, rat, monkey, or zebrafish (cross‐reactivity of an antibody for a target across species). Consider carefully the species used to raise the antibody for the selection of the appropriate secondary antibody that should be directed against, but not raised in, the same species as the primary antibody. Perform a careful titration of antibodies (if possible, using the same batch of antibodies). Provide a clear statement concerning the nature of all the appropriate controls performed for IHC (e.g., 1. documentation or reference to a Western blot confirming an antibody‐antigen binding to detect the target molecule of appropriate molecular size in a cellular lysate; 2). substitution of serum or isotype‐specific immunoglobulins at the same protein concentration as the primary antibody; and 3. genetically engineered controls with PCR confirmation.^18^, ^19^ Include positive and negative tissue controls (in some cases, negative populations within the same specimens that do not express the specific marker can also help to identify positive populations). Consider the differences between humans, mice, rats, and nonhuman primates of the selected protein. Consider specific time‐ and region‐dependent differences of the selected protein.

**TABLE 2:** Key points regarding the identification of cellular components to be considered using in situ hybridization

Use the appropriate brain tissue sample preparation for probes, i.e., frozen and/or formalin‐fixed paraffin‐embedded (FFPE) tissue. Fixation, tissue processing, and target retrieval methods are important elements. A proper selection of the ISH probes. Optimize ISH protocol (e.g., hybridization temperature) for each RNA or DNA probe. Provide a clear statement concerning the nature of all the appropriate controls (e.g., probes with scrambled sequences or genetically engineered controls with PCR confirmation). Consider the differences between humans, mice, and nonhuman primates of the selected mRNA. Consider specific time‐ and region‐dependent differences of the selected mRNA.

### 2.2 Testing and refining consensus by Delphi process^13^

In the first round, 86 statements were evaluated by five working group members. Ninety‐two percent of the votes were in full agreement with the statements, 22% of the votes expressed “no opinion” and 0.6% “disagreed.” The recommendations with undecided and disagreeing votes were discussed once more and revised according to any objections. In the second round all seven group members voted. In the final round, 92 statements found 99% consensus, 1% of statements was voted “no opinion”, and none as “not agreeing”. Based on the discussion after the first voting, two statements had been removed and eight statements were added or detailed from previous ones (Table S1).

## 3 GUIDANCE THROUGH CRFs

### 3.1 Module 1: Basic parameters: Animals and their health status

Please use CRF created by Gorter et al.^5^ (A companion to the preclinical CDEs for physiologic data in rodent epilepsy models. A report of the TASK3 Physiology Working Group of the ILAE/AES Joint Translational Task Force. Epilepsia Open. 2018;3:69–89).

CRF File name: 1 CRF Module‐general health status.docx

CDE File name: 1 General Health Status CDE Chart.xlsx

CRF module: Assessment of the General Health Status of Adult Rodents

***Key points***

- Define the health status of the animal since this may be linked to the neuropathology.
- Standardize procedures for tissue acquisition.

#### 3.1.1 Animals and their health status

The microbiological health status based on the health certificate upon arrival has to be documented. Strain, sex, age, and body weight of the experimental animals should be recorded, as these can have a tremendous impact even without clinical signs. Also, the experimental conditions (e.g., setting of electrodes, electrical stimulation, injection of a convulsant drug, and most importantly a SE) may crucially influence the development of signs of neuropathology (and the outcome of the animal experiment). CDEs presented on the physiology in animal models of epilepsy include a detailed analysis of the health status of experimental animals,^5^ and the reader is encouraged to follow the respective CRFs. As a minimum requirement, we suggest monitoring the age and body weight of the experimental animal and an eventual loss in body weight during the experimental procedure (e.g., surgery and SE), signs of dehydration, possible bleeding from the eyes, and obvious exhaustion or change in apparent behavior (e.g., development of aggressiveness, cognitive deficits, hypoactivity, or isolation in case of group housing). These changes are addressed in CRF‐module 1.

### 3.2 Module 2: Killing and tissue preparation

CRF File name: 2 CRF Module Euthanasia and tissue preparation.docx

CDE File name: 2_CDE_chart_Module_Euthanasia_and_tissue_preparation.xlsx (Supporting information)

***Key points***

- Select an appropriate humane method.
- Collect tissue according to the requirements of the histochemical methods.

The killing of the animal and tissue preparation depends on the subsequent histological or histochemical method applied and should meet international ethical standards (see: https://animal.research.uiowa.edu/iacuc‐guidelines‐euthanasia and https://research.uci.edu/compliance/animalcare‐use/research‐policies‐and‐guidance/euthanasia.html). For obtaining fresh tissue, animals are euthanized by exposing them in a beaker to CO_2_ gas or an alternative method of chemical anesthesia, for example, sodium pentobarbital (ip) or sevofluorane (inhalation like CO_2_). The brains can be dissected into individual brain areas for neurochemical analysis (e.g., radioimmunoassay, Western blotting, etc.) or can be snap‐frozen by insertion into liquid nitrogen or −80°C isopentane (see above) for subsequent ISH, Western blotting, or determination of enzyme activities.

For killing by CO_2_ gas for inhalation, the rodent is placed into a sizeable box (for mice: 16.5 × 30.5 × 14 cm, for rats: 23 × 44.5 × 20.5 cm) not prefilled with CO_2_. The box is then filled with CO_2_ from a gas cylinder or a facility CO_2_ gas distribution system equipped with an appropriate pressure‐reducing regulator and flow meter at flow rates of 4.2 and 12.4 L/min for mice and rats, respectively^20^ (see also: https://rsawa.research.ucla.edu/arc/euthanasia‐rodent/).

For routine IHC or Nissl stain, the deeply anesthetized animals are generally perfused through an intracardial catheter first with buffered saline (for removing erythrocytes which may interfere with the immunohistochemical procedure) and subsequently with the fixative (mostly 4% buffered paraformaldehyde, but also 2% glutaraldehyde or a mixture of these two fixatives). For review and advice see.^21^, ^22^ The animals are decapitated and the brains are removed and postfixed in the fixative for 2–24 h, depending on protocol optimization. The brains can then be snap‐frozen in −80°C isopentane, stepwise freezing going to −80° or in dry ice (2 min). When using isopentane, allow for evaporation of the solvent before sealing the tissue in tubes. Cresyl violet staining is often performed in paraffin‐embedded specimens requiring no cryoprotection.

### 3.3 Module 3: Neurodegeneration: Pathology associated with neurons and neuronal pathways

CRF File name: 3 CRF Module Neurodegeneration.docx

CDE File name: 3_CDE_chart_Module_Neurodegeneration.xlsx (Supporting information)

***Key points***

- Procedures for the neuropathological evaluation are summarized.
- Categories include neurodegeneration, alterations of neuronal morphology, and proliferation.
- Test limitations and optimization of experimental design for evaluation of neuronal alterations in epilepsy research are discussed.

#### 3.3.1 Neurodegeneration

##### Rationale

Neurodegeneration is the term used to describe the progressive loss of structure and function of neurons leading to their death. Neurodegeneration following vascular events, traumatic brain injury (TBI), central nervous system (CNS) infections, neurotoxic drugs, or chronic neurodegenerative diseases may cause seizures and epilepsy, but may also be a pathological consequence of seizures and epilepsy.^23^

Broad scientific evidence, both from clinical and preclinical research, indicates that in adult rats prolonged and recurrent seizures, notably a prolonged SE of one or several hours, induce neuronal death.^24^, ^25^, ^26^ In patients with temporal lobe epilepsy (TLE), a hallmark sign is Ammon's horn sclerosis with different extents of neurodegeneration of individual subfields of the hippocampus.^27^ Mossy cells in the hilus of the dentate gyrus and CA1 pyramidal neurons are highly vulnerable, whereas the subiculum and the granule cell layer of the dentate gyrus are less vulnerable, and TLE pathologies can be classified according to the patterns of losses in individual hippocampal subfields.^27^ It is also important to note that a certain percentage of TLE patients do not show Ammon's horn sclerosis, although a more subtle loss of subclasses of interneurons (e.g., parvalbumin‐containing basket cells) has been observed in the subiculum and sector CA1 of these patients.^28^ Thus, extensive neurodegeneration is not a prerequisite of TLE, although recurrent severe seizures (especially episodes of SE) may facilitate the progression of neurodegeneration and associated neurological symptoms such as cognitive impairment or depression.

Most animal models of epilepsy using an initial SE induced by convulsive drugs, such as kainic acid (KA) or pilocarpine, or by sustained electrical stimulation for inducing recurrent seizures (epilepsy), show similar patterns of neurodegeneration as seen in human Ammon's horn sclerosis.^29^ In addition, other brain areas such as the amygdala, the entorhinal and piriform cortices, and certain thalamic nuclei (e.g., anterior dorsal, anteromedial, and anterior ventral nuclei) may be severely affected.^30^, ^31^ Furthermore, it is important to note that neurodegeneration has been documented in other animal models of epilepsy, especially induced by head trauma.^32^

##### 3.3.1.1 Mechanisms underlying seizure‐related cell death (animal studies)

Studies in animal models have provided insights into seizure‐induced neuronal death and its mechanistic aspects. Notably, sustained SE can be induced by prolonged electrical stimulation^33^, ^34^, ^35^ or injection of neurotoxins like KA,^24^, ^30^, ^36^ bicuculline,^37^ pilocarpine,^35^ or trimethyltin.^38^ SE‐induced cell death in animal models largely resembles Ammon's horn sclerosis seen in TLE patients (although about one‐third of TLE patients does not show such lesions). Importantly, SE‐related neuronal death affects both excitatory principal neurons (pyramidal neurons and mossy cells) and inhibitory interneurons (e.g., hilar somatostatin/neuropeptide Y‐containing interneurons) in the hippocampus.^39^, ^40^, ^41^, ^42^

Besides the hippocampus, SE‐induced neurodegeneration is also seen in the thalamus, olfactory cortex, neocortex, amygdala, and substantia nigra.^30^, ^33^, ^35^, ^43^ Similar patterns of neurodegeneration are observed after ischemia and traumatic brain injury suggesting common underlying mechanisms.^34^ Extensive release of glutamate, induced by the sustained stimulation of glutamatergic neurons during SE, plays a central role. Glutamate operates as an endogenous “excitotoxin,” initiates a massive influx of calcium through NMDA receptor channels, and causes irreversible neuronal injury.^44^

Beyond excitotoxicity, there are other mechanisms leading to neuronal cell death. Bcl‐2 protein family members critically regulate mitochondrial outer membrane permeabilization, an ultimate step in the intrinsic and extrinsic apoptosis pathways.^45^ Apoptosis and possibly other distinct regulated cell death pathways contribute to neurodegeneration following seizures and SE.^46^, ^47^

##### 3.3.1.2 Current classification of cell death types

Cell death has traditionally been identified by studying the morphological alterations of cells. Three morphotypes have been proposed: (1) type I cell death or apoptosis, (2) type II cell death or autophagy, and (3) type III cell death or necrosis.^48^ According to the Nomenclature Committee on Cell Death (NCDD), a clear distinction should be made between accidental cell death (ACD), also called necrosis (the unregulated lysis of cells exposed to severe physical, chemical, or mechanical insults), and regulated cell death (RCD; e.g. apoptosis, autophagy; see below), which relies on dedicated molecular machinery and is potentially modifiable by pharmacological or genetic interventions.^49^ Importantly some forms of RCD occur as part of physiological development and tissue turnover, although perturbations of the intracellular or extracellular microenvironment of sufficient intensity can activate the exact same machinery and lead to cell demise. There is a substantial overlap of the molecular mechanisms involved in the different forms of RCD. Importantly, the view that a distinction can be made between ACD (necrosis) and RCD based on morphology only needs to be revised, as several forms of RCD can present with a necrotic morphotype.^46^, ^50^ For details on the most recent recommendations for the definition and interpretation of cell death from morphological, biochemical, and functional perspectives and classification of RCD by the NCDD we refer to the respective report.^47^ Types of neuronal death studied in epilepsy and other brain injuries are summarized below. For more details about accidental and regulated cell death forms please refer to Galluzzi et al.^47^

##### 3.3.1.3 General procedures for identifying neuronal cell death

###### 3.3.1.3.1 Methods to identify cell death or cell damage without classification

***Nissl stain*** (https://theolb.readthedocs.io/en/latest/histology/cresyl‐violet‐staining‐nissl‐staining.html). In rodent brains, Nissl stain (by staining with cresyl violet) combined with cell counts is most frequently used for identification and (semi)quantification of neuronal cell loss. Cresyl violet binds to basophilic structures like DNA and RNA. It labels not only neurons but also equally stains glia and ependymal cells (see below 2.6.1).

***Fluoro‐Jade stain*** is one of the most commonly used methods to identify acutely injured neurons. There are three Fluoro‐Jade stains available. The newer ones Fluoro‐Jade B and C are preferable since they can be used to detect reliably also dendrites and axons of degenerating neurons.^51^, ^52^, ^53^

***TUNEL stain*** (terminal transferase UTP‐biotin nick end labeling): During apoptosis, DNA is fragmented in a “programmed” mode. The resulting DNA fragments can be labeled at their 3′ ends (end labeling) by reaction with dUTP (or dUTP analogs like BrdUTP or biotin‐dUTP) and terminal deoxynucleotidyl transferase (TT). The dUTP fragments are then identified by IHC in the degenerating neurons or by Western blotting. Using Western blots combined with antibodies for BrdU, DNA laddering (thus the formation of DNA fragments of different lengths induced by programmed cell death) can be shown, whereas histochemistry of, for example, fluorescent‐labeled UTP can label the degenerating neurons, although this procedure allows no final conclusion on the mode of cell death (DNA laddering).^54^ (http://www.ihcworld.com/_protocols/apoptosis/tunel_enzyme.htm).

###### 3.3.1.3.2 Accidental cell death

***Necrotic morphotype***. *Necrosis* refers to accidental cell death characterized by cellular swelling and loss of membrane integrity. It can be studied by electron microscopy to detect the lysis of plasma membrane. It is difficult to diagnose with certainty, as necroptosis, MTP‐driven necrosis and ferroptosis may also have a necrotic morphotype.^47^

###### 3.3.1.3.3 Methods to identify RCD morphotypes according to the underlying mechanism

***Necroptosis***. The regulated form of necrosis necroptosis is triggered by perturbations of extracellular or intracellular homeostasis that critically depends on the receptor‐interacting serine/threonine‐protein kinases RIPK3 and RIPK1, and mixed lineage kinase domain‐like protein (MLKL)^46^ and can also be involved in astrocytic cell death.^55^ It can be studied using immunoblotting and/or IHC for RIPK1, its phosphorylated form p‐RIPK1, RIPK3, and mixed lineage kinase domain‐like protein p‐MLKL (phosphorylated). It can be inhibited with necroptosis inhibitors such as necrostatin‐1.^56^, ^57^, ^58^

***Mitochondrial permeability transition (MPT)‐driven necrosis*** is triggered by perturbations of the intracellular microenvironment and relies on cyclophilin D. It can be studied by defining a necrotic morphology combined with pharmacologic inhibition with cyclophilin D inhibitors (cyclosporin A, sangliferin A, JW47^59^, ^60^).

***Ferroptosis and lipid peroxidation.** Ferroptosis* is considered a new form of regulation of cell death, which is attributed to severe lipid peroxidation caused by the production of reactive oxygen species (ROS) and iron overload found in various neurological diseases, including epilepsy.^61^, ^62^ Ferroptosis is initiated by oxidative perturbations of the intracellular microenvironment that is under constitutive control by the reduced glutathione (GSH)‐dependent enzyme glutathione peroxidase 4 and can be inhibited by iron chelators and lipophilic antioxidants. It can be identified with the help of electron microscopy, Pearl's staining or measurement of iron tissue content, IHC/immunoblot for glutathione peroxidase 4, biochemical measurements of GSH and malondialdehyde, and pharmacologic inhibition with ferroptosis inhibitors (e.g., ferrostatin 1). Lipid peroxidation can be mediated by cyclooxygenases, cytochrome p450, and lipoxigenases (LOXs), among which LOX enzymes drive ferroptosis.^63^

***Apoptotic morphotype***

*Intrinsic apoptosis* is initiated by perturbations of the intracellular or extracellular microenvironment, demarcated by mitochondrial outer membrane permeabilization and precipitated by executioner caspases, mainly caspase 3 (CASP3). Plasma membrane integrity is maintained; there is rapid clearance of fragments by macrophages or other phagocytes. It is triggered by growth factor withdrawal, DNA damage, endoplasmic reticulum stress, ROS overload, replication stress, microtubular alterations, or mitotic defects. Intrinsic apoptosis can be studied by IHC/immunoelectron microscopy/immunoblot to show cytochrome leakage into the cytoplasm, and formation of Apaf‐1 (Apoptotic protease activating factor 1), activation of caspases 9, 3, and 7.^64^, ^65^

*Extrinsic apoptosis* is initiated by perturbations of the extracellular microenvironment that are detected by plasma membrane receptors, propagated by caspase8 (CASP8) (with the optional involvement of mitochondrial outer membrane permeabilization‐MOMP), and precipitated by the executioner caspases, mainly CASP3. It can be studied by means of IHC/immunoblot for CASP8, CASP3 (procaspases and activated caspases), and/or inhibition with CASP8 inhibitor (e.g., z‐IETD‐fmk).^64^, ^65^

***Autophagy***

*Autophagy‐dependent cell death* depends on the autophagic machinery (or components thereof) for lysosomal degradation of excessive or defective macromolecules and organelles. Cytosolic autophagic vacuoles consume the cell. Autophagy occurs in six steps, *initiation, nucleation, elongation, closure, maturation, and fusion*. It can be studied by means of electron microscopy and IHC with the labeling of autophagic vacuoles for LC3.^66^, ^67^, ^68^

***Tauopathy in animal models of epilepsy***

Tauopathy belongs to a different class of neurodegenerative diseases involving the aggregation of tau protein into neurofibrillary tangles (NFTs) or gliofibrillary tangles. Hyperphosphorylation of the microtubule‐associated protein tau and its resultant aggregation into NFT is a pathological characteristic of neurodegenerative disorders like Alzheimer's disease and is known as tauopathies.^69^ Increased tau phosphorylation has been shown in brains after acute seizures in rodents and in TLE patients, and correlates with cognitive decline associated with TLE.^70^, ^71^ The first tau aggregates form in a few nerve cells in discrete brain areas and become self‐propagating and spread to distant brain regions in a prion‐like manner.^71^ IHC using the monoclonal antibody AT8 can be applied to identify tau protein phosphorylated at both serine 202 and threonine 205. Several other monoclonal antibodies raised against defined phosphorylation sites are also commercially available: AT270 (pThr181), AT100 (pSer212/pSer214), AT180 (pThr231), PHF‐6 (pThr231), 1H6L6 (pThr231), and anti‐tau (pSer404).

#### 3.3.2 Markers for labeling functional components of neurons

##### Stains for perikarya

For *postmortem* neuropathological assessment in human tissue, *hematoxylin & eosin (H&E) stain* is a standard procedure. It stains neuronal cell bodies and glial cells but not dendrites or axons. Injured neurons shrink, become eosinophilic due to condensation of mitochondria, and their nuclei become pyknotic due to irreversible condensation of chromatin in the nucleus^72^ (https://www.leicabiosystems.com/knowledge‐pathway/he‐staining‐overview‐a‐guide‐to‐best‐practices/ and: http://www.ihcworld.com/_protocols/special_stains/h&e_ellis.htm).

In rodent brains, *Nissl stain* by staining with cresyl violet is more common although it labels equally also glia and ependymal cells (see above) (https://theolb.readthedocs.io/en/latest/histology/cresyl‐violet‐staining‐nissl‐staining.html). *Hoechst stain* labels equally double‐stranded DNA in nuclei of neurons and glia. After excitation with ultraviolet light (around 350 nm) it emits blue‐cyan light. Thus, it is often used together with immunofluorescence studies. *DAPI (4′,6‐diamidin‐2‐phenylindol) stain* follows the same principle as the Hoechst stain (http://www.ihcworld.com/_protocols/counterstain_solutions/DAPI.htm). It results in blue fluorescent labeling of nuclei of glia and neurons and is often used for double‐ or triple labeling in immunofluorescence studies.

##### 3.3.2.1 NeuN (neuronal nuclei)

NeuN is frequently used as a neuronal marker using IHC. NeuN is the DNA splicing regulator Fox‐3 and is expressed in the nuclei of most postmitotic neurons. While it is a useful marker for pyramidal neurons, it is not expressed by cerebellar Purkinje neurons, olfactory bulb mitral cells, and some interneurons.^73^ Loss of NeuN positive neurons can indicate neuronal cell loss. NeuN is developmentally regulated and is expressed in postmitotic neurons only.

##### 3.3.2.2 Labeling dendrites and axons

Since all methods mentioned above use stains for basophilic structures (generally DNA and RNA), they do not label neuronal processes like axons or dendrites. For this purpose, silver staining, initially developed by Camillo Golgi and later elaborated by Ramón y Cajal, is a valuable method. Variations of this method include Bielschowsky and the Bodian staining procedures and are often used for neuropathological studies.^74^

Microtubule associate protein 2 (MAP‐2) is sometimes used as an immunohistochemical marker preferentially labeling dendrites, neurofilaments (NFs), and neurofilament heavy (NF‐H) subunits are found in the phosphorylated state in normal axons and in the nonphosphorylated state in normal soma and dendrites. They can be distinguished by specific antibodies for anti‐NF‐H phosphorylated (clone SMI 31) and anti‐NF‐H nonphosphorylated (clone SMI 32).

##### 3.3.2.3 Markers for specifically labeling subtypes of neurons

###### 3.3.2.3.1 Markers of glutamatergic neurons

*Vesicular glutamate transporter‐1, 2 (VGLUT1, VGLUT2)*. Since different types of membrane glutamate transporters (excitatory amino acid transporters, EAAT1‐5) are mainly expressed in astrocytes (EAAT1, 2, 3, 5) or restricted to the cerebellum (EAAT4), they cannot serve as specific markers for excitatory neurons. VGLUT1 and VGLUT2 are specifically expressed in glutamatergic neurons. The proteins mediate specifically the transport of glutamate into the synaptic vesicles of the neuron and are therefore enriched in nerve terminals. VGLUT1 or VGLUT2 are valid immunohistochemical markers for glutamatergic nerve terminals. Glutamatergic cell bodies can be identified by ISH for the respective DNAs.^75^

*CaMK II (Ca* ^*2+*^ */calmodulin‐activated protein kinase II)* is preferentially expressed in principal cells and therefore often used as a marker for excitatory neurons.^76^

###### 3.3.2.3.2 Specific markers for interneurons

*General markers for GABA neurons*. GABA‐ergic neurons (interneurons and projection neurons) can be identified by applying IHC for *GABA*, *glutamate decarboxylases* (GAD1 and GAD2), and the *vesicular GABA transporter* (VGAT).^77^ While antibodies for VGAT preferentially label axons and axon terminals, perikarya can be visualized using GABA or GAD antibodies. It is important to note that depending on the enzyme concentration within the individual neuron, not all GABA‐ergic neurons are detected by the GAD antibodies. An alternative may be to use ISH for GADs.

###### 3.3.2.3.3 Markers for subtypes of GABA interneurons

Subtypes of interneurons are often characterized by their content in calcium‐binding proteins or neuropeptides. It is important to note that unequivocal identification of interneuron subtypes would require the specification of neurophysiological and morphological characteristics of the individual interneuron type.^78^ Rough neurochemical characterization of neurons in a brain section may, however, give some valuable insight into neuropathology. With respect to epilepsy, interneurons either containing parvalbumin or somatostatin are of great interest. The calcium‐binding protein parvalbumin is contained in axo‐axonic cells and basket cells of the hippocampus forming somatic or perisomatic synapses on pyramidal cells, whereas the neuropeptide somatostatin is contained (among others) in interneurons innervating principal cell dendrites, like O‐LM cells of the hippocampus. Both cell types can undergo neurodegeneration in epilepsy. Other interneuron types contain the neuropeptides cholecystokinin‐octapeptide (CCK), neuropeptide Y (NPY), vasoactive intestinal polypeptide (VIP), or neurokinin B (NKB) or can be labeled by the calcium‐binding proteins calretinin and calbindin, or by the enzyme nitric oxide synthetase (NOS). This is only a selection of a continuously increasing list of interneuron‐specific markers.

##### 3.3.2.4 Markers for neuronal activation: cfos, ΔFosB, and Arc

One of the most widely used methods to monitor neuronal activation by histochemistry is the 2‐deoxyglucose method by Sokoloff.^79^ It is based on the accumulation of radioactive 2‐deoxyglucose that uses glucose uptake into neurons but is not or only slowly metabolized compared with glucose. This requires treatment of the animals with radioactive ^14^C‐2‐deoxyglucose and subsequent autoradiography. More recently, ISH or IHC for the immediate early genes *cfos* and ΔFosB have been introduced.^80^ These methods are based on the fast expression of the immediate early genes upon neuronal stimulation (e.g., seizures). Expression of *cfos* is fast but only short‐lasting with a maximum after about 2 h, whereas ΔFosB immunoreactivity can be detected even after weeks in the stimulated cells (neurons but also astrocytes). Another immediate early gene used for identifying activated neurons is *Arc*.^81^ *Arc* mRNA is already expressed after a few minutes and has a half‐life of about 45 min.

##### Markers for proliferating neurons in the hilus of the dentate gyrus

In the subventricular zone and in the subgranular zone of the dentate gyrus, progenitor cells are present that can divide and differentiate into neurons. Seizures are promoting adult neurogenesis.^82^ Proliferating neurons or glia can be identified by injecting *BrdU*, ip, into the animals. BrdU is incorporated into the DNA during the S‐phase of dividing cells and can be identified ex vivo by IHC. Proliferation, differentiation, and maturation of “newly born” cells/neurons are linked to the sequential expression of different factors, like *PCNA* (proliferating cell nuclear antigen), *Ki67, nestin, doublecortin (DCX), calretinin*, and many others, which are often used for further characterization of the cells. Ki‐67 is thought to represent the most reliable marker for cells that reenter the cell cycle. It is expressed during different phases of the cell cycle and therefore labels more dividing cells than BrdU.^83^, ^84^ In late postmitotic phases of neurogenesis, new neurons express calretinin and DCX transiently together with *NeuN*,^84^, ^85^ whereas mature granule cells then express calbindin and NeuN but not calretinin.^84^ Taken together, the most reliable methods for identifying “newly born” neurons or glial cells are the BrdU method and IHC for Ki‐67.^83^

### 3.4 Module 4: Pathology related to astrocytes

CRF File name: 4 Astrocyte pathology.docx

CDE File name: 4_CDE_chart_Module_Astrocyte_pathology.xlsx (Supporting information)

***Key points***

- Categories include alterations of astrocytes such as morphology, degeneration, and proliferation.
- Test limitations and optimization of experimental design for evaluation of astrocyte alterations in epilepsy research are discussed.

#### 3.4.1 Rationale and methods

##### Astrocyte morphology

Astrocytes (“star‐like cells”) are quite diverse, and the term astrocyte or astroglia encompasses multiple types of structurally and functionally distinct cells. The more that is being learned about astrocytes, the more it is clear that there is great structural and functional heterogeneity among astrocytes, both within and between distinct brain regions.^86^, ^87^ Nevertheless, it is useful to briefly review the main features of astrocytes. The largest morphological subdivision has been between *protoplasmic astrocytes* and *fibrous astrocytes*. Protoplasmic astrocytes, found in the gray matter of the brain and spinal cord, have several (5‐10 in rodent) primary processes which then expand into a complex arborization. Interestingly, this arborization delineates a “domain” and in the normal state there is little overlap between astrocyte domains.^88^, ^89^ Each protoplasmic astrocyte extends processes to ensheathe synapses (perisynaptic processes),^90^ contact blood vessels (forming a specialized structure called a perivascular endfoot), and sometimes the pial surface (subpial endfeet, collectively forming the *glia limitans*). The processes from a single rodent protoplasmic astrocyte contact approximately 100,000 synapses.^88^, ^91^ Human astrocytes in the neocortex are much larger and more complex than rodent astrocytes,^92^, ^93^ although species‐dependent differences in morphology and function are less obvious in other brain regions.^94^

Fibrous astrocytes, found in the white matter of the brain and spinal cord and in the optic nerve and nerve fiber layer of the retina, are less arborized than protoplasmic astrocytes, and their cell bodies are located in a regular pattern between the axon bundles. In addition to perivascular and/or subpial endfeet, they also extend perinodal processes to contact the axons at nodes of Ranvier.^95^ While fibrous astrocytes exhibit diverse morphology, in general they lack domain organization.^96^

Originally, it was thought that astrocytes could be segregated by molecular markers, notably by expression of the intermediate filament protein glial fibrillary acidic protein (GFAP). However, GFAP expression is heterogeneous among astrocyte subtypes. For example, fibrous astrocytes express GFAP to a greater extent than protoplasmic astrocytes, and there is the heterogeneity of GFAP expression among protoplasmic astrocytes of distinct brain regions. Astrocyte heterogeneity of form and function is the rule rather than the exception.^86^, ^87^, ^97^

##### 3.4.1.1 Heterogeneity of astrocytes

The application of more sophisticated research strategies, together with a systematic evaluation and comparison of different glial markers, represent the best approach toward new advances in the identification of astrocyte subpopulations, specific markers, and a better understanding of their role in brain function under both physiological and pathological conditions. Large‐scale gene expression studies (including more recently RNA‐Seq‐based transcriptomes from region‐specific brain samples and specific cell types) provided a global characterization of the astrocyte transcriptome and the identification of cell‐type‐specific markers. These studies have identified a number of genes that are highly enriched in astrocytes, compared with other neural cells (neurons and oligodendrocytes); these astrocyte‐enriched genes include, besides GFAP, a variety of extracellular secreted proteins, glutamate transporters (e.g., GLT1 and GLAST), enzymes, receptors/membrane proteins (e.g., connexin43), and transcription factors.^98^, ^99^

Single‐cell transcriptomic studies provide additional strong evidence of the spatial and temporal heterogeneity of astrocytes, revealing morphologically and physiologically distinct astrocyte subtypes between and within brain regions.^100^, ^101^

##### 3.4.1.2 Astrocytes in epilepsies: Changes in morphology and proliferation

One striking hallmark of the sclerotic hippocampus is that while there is a specific pattern of neuronal loss, there is also “reactive gliosis” with hypertrophic glial cells exhibiting prominent GFAP staining and long, thick processes. In contrast to all the studies of hippocampal neuron loss, only a few studies have attempted to quantitate changes in astrocyte numbers and densities in epileptic tissue.^102^, ^103^, ^104^, ^105^ Previously, it was believed that neuronal loss led to astroglial phagocytosis and, consequently, increased gliosis.^106^ This view has changed, however, as a reactive change in astrocytes, or *reactive gliosis (astrogliosis)*, is commonly found in both sclerotic and nonsclerotic hippocampal tissue specimens. Gliosis commonly involves fibrillary gliosis in CA1 and radial gliosis in the dentate gyrus.^107^ These glial cell changes have become a hallmark of the sclerotic hippocampus and involve astrocyte hypertrophy,^102^, ^108^, ^109^, ^110^, ^111^, ^112^ increased expression of GFAP^112^ and vimentin,^113^, ^114^, ^115^ and changes in glial‐specific proteins.^108^, ^113^, ^115^ Thus, it is becoming increasingly clear that multifaceted changes in astrocyte phenotype may play more of a causative role in the overall pathology and seizure susceptibility.^106^, ^108^, ^116^, ^117^, ^118^ One clinical report indicates a positive correlation between chronic seizure burden and degree of gliosis, and interestingly an increase in reactive astrocyte number in CA3 was the strongest predictor of poor postoperative seizure outcome.^119^

Astrocyte morphological changes in human hippocampal sclerosis specimens and in rodent models of epilepsy have largely been defined with GFAP immunoreactivity. In epileptic specimens in post‐SE models of epilepsy, astrocytes adopt a “reactive” phenotype in which they become hypertrophic with long, thick processes and increased GFAP immunoreactivity.^120^, ^121^ In the chronic phase, astrocytes in hippocampal specimens from TLE patients or some TLE mouse models display striking morphological features that are clearly distinct from their nonsclerotic counterparts.^116^, ^122^, ^123^ However, the use of GFAP immunoreactivity alone to examine changes in astrocyte morphology has significant limitations. As noted above, GFAP expression is heterogeneous among astrocyte subtypes, and within individual astrocytes GFAP tends to label primary and secondary processes but not the entire astrocytic “tree” or domain. S100β is another well‐known astrocytic marker, but it undergoes activity‐dependent release,^124^ which may undermine its ability to serve as a reliable morphological marker. Astrocytes in epileptic tissue have also been identified by staining with astrocyte‐specific markers such as GLT‐1 (Na^+^‐dependent glutamate transporter) and AQP4 (aquaporin‐4 water channel). However, while predominantly astrocytic, GLT‐1 is also present on neuronal axon terminals.^125^ It has also been found that both GLT‐1^126^, ^127^ and AQP4^120^, ^127^ are regulated in epilepsy and therefore cannot independently serve as morphological markers to evaluate changes in astrocyte morphology.

Thus, available histochemical markers may be insufficient to fully characterize astrocyte morphological changes in epilepsy. Interestingly, evaluation of the entire astrocytic “domain” has demonstrated that there may be a loss of astrocyte domain organization in animal models of epilepsy.^128^ To assess domain organization and morphological changes in astrocytes in more detail in rodent epilepsy models, genetically targeted mice with brightly fluorescent astrocytes (e.g., Aldh1l1‐EGFP mice) are available (https://www.jax.org/strain/026033). Such mice could potentially be used in future studies to more carefully define astrocyte morphological changes during epileptogenesis, obviating the drawbacks of histochemical stains. In addition, changes in protoplasmic versus fibrous astrocytes in epilepsy models have not been fully assessed.

In addition to morphological changes in existing astrocytes, the proliferation of astrocytes has also been described as a pathological hallmark in human epilepsy tissue specimens.^102^, ^103^, ^104^, ^105^ However, astrocyte proliferation has not yet been well studied in many rodent models of epilepsy. Mitotic labeling with Ki‐67 and double‐labeling with GFAP and/or with fluorescent astrocytes would possibly enable the determination of the degree of de novo astrocyte proliferation at various time points during epileptogenesis in rodent models of epilepsy. Some studies have indeed shown “proliferating” astrocytes with double‐labeling with GFAP and Ki‐67.^129^, ^130^ However, in most studies, the production of new astrocytes (proliferation) has not been distinguished from morphological changes in existing astrocytes. This should also be a topic of future studies to more fully evaluate phenotypic changes occurring in astrocytes during epileptogenesis in animal models. Of course, such studies will need to take into account the resident stem cell population in the subgranular zone (SGZ) of the dentate gyrus and its proliferative response in seizures and epilepsy independent of changes in the proliferation of existing astrocytes.^131^, ^132^, ^133^ Similarly, SE may induce proliferation of other glial cell types such as NG2 cells.^134^ Thus, “gliogenesis” during epilepsy will need to be carefully defined in each model both by cell type and by region. Finally, genomic analysis of reactive astrocytes in epilepsy models (as has been done for ischemia and neuroinflammation^135^) could yield new pathophysiological insights. Thus, earlier studies using transcriptomics suggested differentiation of astrocytes into an A1 and A2 phenotype assisting classification of neurotoxic and neuroprotective alterations during pathophysiological conditions, respectively. However, meanwhile, there is increasing evidence that astrocytes adopt multiple additional molecular and functional states during pathogenesis.^136^

##### 3.4.1.3 Astrocytes in epilepsies: Degeneration in epilepsy

Recent data suggested that altered functional properties of astrocytes might contribute to or even cause TLE,^116^, ^137^ but whether or not and through which mechanisms astrocytes may also undergo cell death during epileptogenesis is a matter of intense investigation. Previous work has reported astrocytic degeneration in the hippocampus, dentate gyrus, or neocortex after pilocarpine or KA‐induced SE.^58^, ^138^, ^139^, ^140^, ^141^, ^142^, ^143^ These animal model‐based studies reported a species‐dependency (mouse vs. rat) and concluded that astrocytes preferentially die at later stages (days to weeks) after epilepsy induction through autophagy, apoptosis, or necroptosis (regarding classification of cell death types, see Module 3 of this article). During or post‐SE, the energy reserves of the cells run out, which may induce autophagy.^144^ Autophagy in astrocytes may also be induced by TNFα following SE,^145^ and data obtained from neurosurgically resected specimens from TLE patients show increased TNFα levels.^146^ Interestingly, necroptosis, a regulated form of necrosis, is also induced by TNFα.^46^

Whether astrocytes die already immediately (a few hours) after induction of SE has been addressed by only a few studies to date. Using a unilateral intracortical KA injection model of TLE,^116^ did not find TUNEL‐ or Fluoro Jade‐positive apoptotic astrocytes 4 h after KA injection. These findings were recently confirmed in the same model where the same group instead found evidence for early activation of autophagic (LC3B) and necroptotic (RIPK3, MLKL, and pMLKL) markers in astrocytes of the ipsilateral hippocampus.^55^ Since hippocampal TNFα is enhanced within a few hours post‐SE in this model,^147^ the data suggest that this cytokine plays a key role in astrocytic cell death occurring immediately after SE induction.

##### 3.4.1.4 Methodical pitfalls

Some methodical caveats have to be considered. First, S100β and GFAP are two widely used markers for astrocytes.^148^ However, S100β is released by astrocytes by various stimuli, which in turn influences neuronal activity.^149^ Interestingly, in acute hippocampal slices, KA potentiated the release of S100β in a neural‐activity‐dependent way,^124^ which might explain the loss of S100β immunoreactivity after KA‐induced TLE in vivo.^150^ It is therefore essential to use other/additional cell type‐specific marker(s), for example, double staining for GFAP and a nuclear marker, before concluding about the potential loss of astrocytes. Second, during epileptogenesis, caspase‐3 activation seems to have a dual role in astrocytes. In a focal cortical dysplasia rat model, it (i) indicated cytoskeletal remodeling in astrocytes early (3–5 days) after pilocarpine‐induced SE while (ii) denoting astroglial death only at late stages (several months after SE) as evidenced by costaining with Fluoro‐Jade.^143^ Thus, detection of caspase‐3 alone is insufficient to prove astrocyte death.

### 3.5 Module 5: Epilepsy‐induced immune responses

CRF File name: 5 CRF Module Immune responses.docx

CDE File name: 5_CDE_chart_Module_Immune_responses (Supporting information)

The following information is provided to facilitate the use of the CRF for data acquisition. The CRF may be modified according to the choices made for obtaining data, as discussed below.

***Key points***

- Procedures for the neuropathological evaluation are summarized to accompany preclinical CDEs.
- Categories include innate immune response and adaptive immune response.
- Test limitations and optimization of experimental design for evaluation of the immune response in epilepsy research are discussed.

#### 3.5.1 Rationale and methods

##### Immune response

A large body of evidence that has accumulated over the past decade (obtained in animal models of epilepsy and human brain specimens from various drug‐resistant forms of epilepsy) strongly supports the role of both innate and adaptive immunity in the pathophysiology of epilepsy (reviewed in Refs. [151, 152, 153, 154, 155]). In particular, evidence from experimental models of TLE (models of post‐SE evoked by electrical stimulation or chemoconvulsant drugs, models in rats or mice) and TBI models demonstrated a common innate immunological response of the CNS, involving microglia and astrocyte activation, with the production of various inflammatory molecules. Moreover, there is increasing evidence (from experimental models and human studies) supporting the contribution of inflammatory mechanisms to the neurological manifestations of genetic epilepsies, such as genetic absence epilepsy, tuberous sclerosis complex, and other related epileptogenic developmental pathologies.^156^, ^157^, ^158^, ^159^, ^160^, ^161^ If the experimenter decides to include the evaluation of the immune response parameter in the experimental design, it is essential to report the immunological status of the animals (specific pathogen‐free or conventional condition; individual vs group housing) as well as considering the immunological differences between species and sex in the interpretation of an IHC assay. Next, it is recommended to evaluate the principal components of both innate and adaptive immunity with the proper selection of antibodies, techniques (fixation, tissue processing, immunoreactions and antigen retrieval methods, and adequate controls). Our description of the neuropathological evaluation of the immune response aims to assist the investigators in the exploration of the immune system in models of epilepsy and is not intended to provide a comprehensive manual or review, but rather a reference guide to be used in association with the CDEs.

##### 3.5.1.1 Innate immune response

The innate immune response is a nonspecific, acute defense against external agents or local injuries, and cell types involved in this response include microglia, monocytes/macrophages, and also astrocytes.^162^

###### 3.5.1.1.1 Microglia

Microglial cells are highly specialized and dynamic resident immune cells in the CNS that constitute the key innate immune cell in the CNS. Microglial cells represent approximately 10% of the total number of cells within the adult CNS, with different microglial densities in distinct brain regions and recent evidence of spatial and temporal heterogeneity of both rodent and human brain microglia.^163^, ^164^, ^165^ The detection of microglial cells is usually based on their morphological features (including changes in cell body size, process length, process numbers, and complexity of branching) together with immunohistological stains, using classical activation markers such as ionized binding adaptor molecule‐1 (Iba‐1), Cluster of Differentiation 68 (CD68), or CD11b.^146^, ^154^, ^166^ Lectin histochemistry (lectin‐horseradish peroxidase stain) and Iba1 are most suited for structural studies to visualize especially the ramified microglia. Lectin histochemistry recognizes sugar residues also in endothelial cells, thus it can be useful for the study of the relationship between microglia and vasculature.^167^, ^168^ CD11b and CD68 can best be used when studying activated microglia. The macrophage scavenger receptor CD163 can be used to identify perivascular macrophages, as well as populations of meningeal and choroid plexus macrophages. Microglia share many surface markers with peripheral tissue macrophages, which can also infiltrate the brain in epilepsy models associated with BBB dysfunction (e.g., models of post‐SE and TBI^169^; and can be important to distinguish microglia from other myeloid cells. Approaches based on morphological distinctions (ramified vs. amoeboid) or relative marker expression of the common leukocyte antigen CD45 have limitations in the context of epilepsy models (morphology and CD45 expression change with inflammation or injury). The fractalkine receptor CX3CR1 is also highly expressed in activated microglia^170^ and can be detected in circulating monocytes and other myeloid cells. Microglia express also receptors for ATP (purinergic receptors, such as P2X4, P2Y6, P2X7, and P2Y12), involved in the regulation of neuron‐microglia interactions.^171^ Recently, the transmembrane protein 119 (Tmem119) has been identified as a potential microglia‐specific marker in both the mouse and human brain.^172^

Similar to the broad spectrum of reactive astrocytes, activation of microglia also displays a continuum of pathological changes ranging from the proinflammatory, potentially cytotoxic M1‐ to the antiinflammatory, scavenging, and regenerative M2‐type microglia.^173^ Jurga et al. provide a comprehensive overview of currently anticipated microglia dysfunctions and markers at different pathological states. Importantly, several studies also demonstrated species‐specific patterns of gene expression and different responses of human versus rodent microglia under pathological conditions. Thus, caution should be taken while choosing appropriate markers for microglia activation.^174^, ^175^ For studying microglia subpopulations low/mass cytometry can be also used with the above‐mentioned caution with respect to differences between different species, as well as regional heterogeneity.^176^ Recently emerging single‐cell techniques, such as cytometry by time‐of‐flight mass spectrometry (CyTOF) and single‐cell RNA sequencing, and their combination may help researchers to overcome such limitations and guide in the selection of the appropriate markers reflecting the pathology‐dependent heterogeneity of microglia in different experimental conditions.^177^, ^178^

###### 3.5.1.1.2 Astrocytes

Astrocytes are known to play a major role in the regulation of the immune/inflammatory response in several human CNS diseases, including epilepsy.^154^, ^162^ Extensive information concerning the classical astrocytic markers has been provided within the CRF module focusing on this cell type and can be used to identify astrocytes and their morphological and functional changes also in relation to their role in the immune response. Astrocytes represent an important source of immunologically relevant cytokines and chemokines, components of the complement pathway and they are also the target of inflammatory molecules which, through the activation of specific receptors (including pattern‐recognition receptors and related intracellular signaling pathways, such as IL‐1 receptor/toll‐like receptor‐mediated pathways; complement receptors, etc.) may aggravate astrogliosis and amplify the proepileptogenic inflammatory signaling.^154^, ^162^ In the evaluation of the astrocyte‐mediated immune response in experimental models, it is important to consider that human and rodent astrocytes share many similar properties,^94^ although differences in their genomic and functional features have been found in cell culture.^179^

##### 3.5.1.2 Adaptive immune response

###### 3.5.1.2.1 T‐ and B‐lymphocytes

Experimental studies and clinical evidence obtained in animal models of epilepsy and human brain specimens from various drug‐resistant forms of epilepsy have demonstrated also the activation of adaptive immunity mechanisms.^32^, ^146^, ^151^, ^180^, ^181^ This response can also be maladaptive and react to self‐antigens, thus resulting in autoimmunity. The adaptive immune response is antigen‐specific, and T‐ and B‐lymphocytes are the key players in adaptive immunity. IHC for CD20 (which is expressed at all stages of B‐cell development) can be used to determine the presence of B‐lymphocytes in histological tissue sections. An interesting subpopulation of CD20‐positive B‐cells is represented by the regulatory B (Breg), which participates in immunomodulation and in suppression of immune responses. Breg cells express IL‐10 (but also other cytokines such as TGF‐β and IL‐35) and have been named B10 cells. To date, there is still discussion about the specific Breg phenotype; thus a combination of several markers is usually required and the best combination of markers selected may vary in different tissues and species or on the degree of inflammation.^182^, ^183^

A cluster of differentiation 3 (CD3; expressed at all stages of T‐cell development) is the commonly used immunohistochemical marker for T‐cells in tissue sections. To study in more detail the infiltration of T‐lymphocytes into CNS, T‐lymphocytes, CD8+ cytotoxic T (Tc), and CD4+ T‐helper (Th) lymphocytes can be further identified. These lymphocyte markers (CD20, CD3, CD4, and CD8) have been already used both in animal models of epilepsy and human brain epilepsy specimens using IHC^146^, ^166^, ^180^ or flow/mass cytometry.^184^, ^185^ Cytotoxic lymphocytes (as well as natural killer cells) utilize the perforin/granzyme pathway for target‐cell killing, thus antibodies antiperforin or granzyme B, could be also included in the evaluation of cytotoxic T‐cells.

An interesting subpopulation of CD4+ T‐cells is represented by the regulatory T‐cells (Tregs: thymus‐derived Tregs and peripherally derived Tregs), which may control the immune response.^186^ Several antibodies have been introduced to identify and characterize the Treg populations (such as CD25, FoxP3, and CD127), allowing better identification of the Tregs population. The minimum panel to characterize Treg cells may include: CD3, CD4, CD25, and FoxP3, however, the best combination of markers selected by the investigator may depend upon the tissue and species or the degree of inflammation.^186^

##### 3.5.1.3 Innate or adaptive immunity crosstalk: Dendritic cells, natural killer, inflammatory mediators

###### 3.5.1.3.1 Dendritic cells

Dendritic cells (DCs) represent a heterogeneous group of cells with a strong antigen‐presenting capacity, acting as sentinels of the immune system. In the CNS under physiological conditions, DCs are strategically located in the choroid plexus, meninges, and perivascular spaces.^187^, ^188^ Recruitment of DCs in brain tissue has been shown in Li‐pilocarpine induced SE model, using CD11c (a pan DCs marker).^189^ Other DC markers, such as CD83 and DC‐specific ICAM‐3‐grabbing nonintegrin (CD209) have been also used to evaluate the presence of DCs in the epileptogenic brain and rat tissue.^166^, ^180^

###### 3.5.1.3.2 Natural killer cells

Natural killer (NK) cells represent a component of the innate immune system acting as first effectors during the earliest phases of immune response and represent key players in both the innate and adaptive immune responses via their cytolytic activity and through cytokine production. They constitute a population of CD3 negative innate lymphoid cells that can be rapidly mobilized and have been shown to be recruited to the CNS following several pathological conditions.^190^ NK display a large variety of phenotypes and functions and the local microenvironment may influence the tissue‐specific properties of NK cells,^191^ complicating the study of these cells in experimental animal models (for detailed information concerning NK phenotypes and most common NK markers, such as CD16, CD56, and NKp46, see^190^, ^192^, ^193^). Recruitment of NKs has been recently shown in brain tissue from pediatric epilepsy patients.^184^, ^185^

###### 3.5.1.3.3 Inflammatory mediators

Communication between the innate and adaptive responses involves cell‐cell interactions, as well as soluble factors such as cytokines, such as interleukin(IL)‐1β, IL‐6, tumor necrosis factor (TNF)‐α, transforming growth factor beta (TGF)‐β, and chemokines, such as monocyte chemoattractant protein‐1 (MCP‐1; chemokine, C‐C motif, ligand 2; CCL2), components of the complement pathway, such as C1q, C3, and other molecules and related receptors studied in both experimental and human epilepsy. For extensive information concerning the inflammatory mediators identified in animal models of epilepsy and human epileptogenic tissue, the reader is referred to specialized reviews on the topic.^152^, ^154^, ^155^

### 3.6 Module 6: Oligodendrocytes, myelin, and BBB

CRF File name: 6 CRF Module Oligodendrocytes, myelin and BBB.docx

CDE File name: 6_CDE_chart_Module_Oligodendrocytes_and_myelin.xlsx (Supporting information)

#### 3.6.1 Oligodendrocytes and myelin

***Key points***

- Methods for investigating myelin in epilepsy are presented.
- Markers for oligodendrocytes are included.

##### 3.6.1.1 Rationale and methods

Oligodendrocytes electrically insulate neuronal axons by wrapping of concentric sheaths of myelin around them and thereby allow rapid signal transmission (saltatory neurotransmission). Moreover, they also provide metabolic support to neurons, for example, by providing lactate to neurons for ATP generation.^194^, ^195^, ^196^, ^197^ During brain development, oligodendrocyte precursor cells are able to dynamically respond to the needs of the developing networks, but myelin remodeling also continues throughout adulthood. Only in recent years it has been shown that myelination is sensitive to neural activity and altering myelin structure can affect behavior, thereby representing a novel form of plasticity in the CNS.^198^, ^199^ There is evidence that seizures can affect oligodendrocytes and the myelin sheath.^200^, ^201^ MRI and DTI studies suggested structural and functional white matter abnormalities in epilepsy patients,^202^, ^203^, ^204^, ^205^ and these abnormalities may correlate with impaired cognitive and emotional functions such as language processing and working memory.^206^, ^207^, ^208^

In studies on autoimmune encephalitis and multiple sclerosis, a great variety of antibodies to myelin proteins have been created and can serve as markers for oligodendrocytes and myelin.^209^ Among these are 2′,3′‐cyclic‐nucleotide 3′‐phosphodiesterase (CNPT), a marker of immature and mature oligodendrocytes (2 days postinjury), myelin basic protein (MBP; 7 days to 3 months postinjury), and myelin oligodendrocyte glycoprotein (MOG), oligodendrocyte lineage transcription factor 2 (olig2), and Luxol fast blue stain (LFB stain).^210^ Noteworthy, olig2 was observed also in astrocytes of the thalamus^97^ and in NG2‐positive cells,^211^ suggesting some caution when used as oligodendrocyte marker.

#### 3.6.2 Changes in brain vasculature and BBB in epilepsy

***Key points***

- Opening of the BBB can be an early consequence of seizures but can also have a significant impact on the development of epilepsy.
- Procedures for investigating the opening of the BBB are presented.
- Methods for investigating components of the microvasculature (epithelial cells, pericytes) are presented.

##### 3.6.2.1 Rationale

Epileptiform activity and notably SE can cause disruption of the BBB, plasma extravasation, and formation of brain edema^169^, ^212^, ^213^, ^214^ and subsequent remodeling of the vasculature.^215^ For demonstrating an opening of the BBB, rodents can be injected with Evans blue or fluorescein‐albumin. Evans blue binds to albumin and thus, like fluorescein‐albumin, does not cross the BBB. Both compounds can be identified in the brain tissue after killing and perfusion of the animal by the blue color or fluorescence, respectively.^212^, ^213^ Alternatively, horseradish peroxidase can be infused as a marker and identified by the peroxidase‐antiperoxidase reaction, or endogenous albumin that has migrated to the tissue can be visualized by immunocytochemistry.^212^ Opening of the BBB and resulting brain edema can also be detected in the living animal by magnetic resonance imaging (MRI).^216^

##### 3.6.2.2 Methods

*Opening of the BBB* in epilepsy is intimately linked to a remodeling of the vasculature.^217^ Altered vasculature, the opening of the BBB and brain edema have been postulated to be causative for the development of epilepsy already in the early 20th century.^218^ Remodeling of the vasculature may be driven by vascular epithelial growth factor (VEGF, expressed in astrocytes) and its receptor VEGFR‐2.^215^, ^217^

***Blood vessels*** are composed of two main cell types: endothelial cells, which form the lumen of blood vessels, and mural cells (vascular smooth muscle cells and pericytes). In the brain, endothelial cells are connected by tight junctions and enwrapped by pericytes in the microvasculature. Pericytes are critical for the development of the BBB and regulate blood flow in response to neuronal activity and neurotransmitter release in adulthood. Conversely, pericyte dysfunction or loss may contribute to neurodegenerative diseases. Antibodies for CD31 (platelet/endothelial cell adhesion molecule‐1), Tie2 (tyrosine kinase 2, the receptor for angiopoetin 1),^219^ and CD34, and the monoclonal RECA‐1 antibody specifically directed to a surface protein of endothelial cells^220^ and others (e.g., plant lectin Ulex europaeus agglutinin‐I, UEA‐I) is used as a marker for endothelial cells and for the vasculature in the brain. On the other hand, proteoglycan NG2 and platelet‐derived growth factor receptor‐β (PDGFRb) and aminopeptidase N (CD13) can serve as markers for pericytes.^134^, ^221^ Selective labeling of arterioles: Arterioles can selectively be labeled by local intracerebral or intravenous injection of Alexa‐Fluor 633 hydrazide and identified by fluorescence microscopy in the subsequently fixed tissue.^222^

## 4 DISCUSSION AND CONCLUSION

The underlying pathophysiology is an important aspect of understanding epilepsies and for developing rational therapies. With the CRF forms included in the present article, we aimed to give an overview and guidelines for a rational analysis of the pathophysiology of epilepsy models using biomarkers for detecting neuropathological changes during epileptogenesis or following manifestation of epilepsy. In practice, however, only a selection of markers may be crucial for individual studies with defined goals. Thus, neurodegeneration (e.g., assessed by Nissl staining), activation of astrocytes and microglia (assessed, e.g., by GFAP and CD11 IHC, respectively), or activation of neuronal units (ΔFosB IHC) may be crucial general parameters for pathophysiological events. They should be followed by a more detailed analysis of, for example, the mechanisms of neurodegeneration and inflammation, by investigating individual markers for autophagy or apoptosis, or the type of inflammation or expression of individual cytokines.

Similarly, altered morphology, for example, of neurons or astrocytes, may provide insights into pathophysiological changes during epileptogenesis. Thus, Ammon's horn sclerosis *per se* reflects a dramatic rearrangement of the hippocampal circuitry. Determining changes in the expression of specific markers for interneurons (e.g., GAD1, neuropeptides such as somatostatin or calcium‐binding proteins like parvalbumin) or principal cells (VGLUT1) may depict either specific neuronal loss or over‐expression of the respective parameter, often reflecting activation of specific neurons. Rearrangement of neuronal circuitries may also include sprouting of certain neuronal pathways as often indicated by condensation of immunoreactive axon terminals. Most spectacular examples are mossy fibers sprouting to the inner molecular layer of the dentate gyrus in rats with hippocampal sclerosis^223^ or sprouting of (somatostatin) interneurons in the middle and outer molecular layer of the dentate gyrus^224^ and the outer molecular layer of the subiculum.^225^ It is often unclear whether such mechanisms support seizure activity, or represent endogenous antiepileptic mechanisms (or both). A pathological sign often seen in humans is granule cell dispersion, sometimes presented by a double layer of granule cells.^226^ In animal models using intrahippocampal or intracortical injection of pilocarpine or KA, a similar picture of progressing broadening and dispersion of the ipsilateral granule cell layer can be observed.^116^, ^121^ The pathophysiological relevance and underlying mechanisms are still not entirely clear.

In the same way as the rearrangement of neuronal circuitries, processes including astrocytes and inflammatory processes are fundamental in the development of epilepsy. In particular, brain injury or proconvulsant events can activate microglia and astrocytes to release a number of proinflammatory mediators, thus initiating a cascade of inflammatory processes in brain tissue. Proinflammatory molecules can affect the physiological functions of glia, alter neuronal excitability and perturb glio‐neuronal communications.^116^, ^151^ In the same way, the opening of the BBB is an early pathophysiological sign during a SE but by itself also strongly contributes to seizure generation.

It is important to emphasize that neuropathological examination always has to be accompanied by a careful behavioral and physiological examination of the animals to be meaningful. Animal models are thought to mirror the situation in human epilepsy patients. Since there exists a significant number of different human epilepsies, the choice of the appropriate animal model is crucial to match the human disease. Fortunately, many of the observations obtained in animal models match the results from human tissue. One should, however, also consider potential differences in the pathology between animal models and epilepsy patients and be aware of species differences between tools like antibodies or probes for ISH.

In conclusion, the present paper together with CDEs and CRFs aims to be a guideline for neuropathological examination in experimental models of epilepsy. Needless to say, in practice, only a subset of the presented neuropathological methods will be applied. The use of CDEs, however, may help to standardize these neuropathological examinations and will allow comparison between different studies finally allowing the performance of homogeneous metaanalyses and rational target‐oriented drug studies.

## CONFLICT OF INTEREST

None of the authors has to disclose a potential conflict of interest.

## ETHICS STATEMENT

We confirm that we have read the Journal's position on issues involved in ethical publication and affirm that this report is consistent with those guidelines.

## Supporting information


TABLE S1

Appendix S1

## Appendix

**Immunohistochemistry:**

We refer to the following two Handbooks for guidance on immunohistochemical methods. You can find their details about different immunohistochemistry methods (secondary abs, chromogens like DAB or NovaRED, multiple immunoenzyme staining, quantitative image analysis).

***Handbook of Practical Immunohistochemistry**, Eds.: Fan* Lin and Jeffrey Prichard, *Springer (ISBN 978‐1‐4939‐1578‐1)*

***Immunohistochemistry: Basics and Methods***, by Igor B. Buchwalow and Werner Böcker, *Springer (ISBN 978‐3‐642‐04609‐4)*

***Immunohistochemistry, Essential Elements and Beyond***, by Alexander E. Kalyuzhny*, Springer (ISBN 978‐3‐319‐30893‐7)*

**Links to methodological explanations/protocols by some commercial suppliers of antibodies:**

http://www.ihcworld.com/_books/Dako_Handbook.pdf

https://www.thermofisher.com/at/en/home/life‐science/protein‐biology/protein‐biology‐learning‐center/protein‐biology‐resource‐library/pierce‐protein‐methods/overview‐immunohistochemistry.html

https://www.bosterbio.com/protocol‐and‐troubleshooting/immunohistochemistry‐ihc‐principle

https://www.leicabiosystems.com/knowledge‐pathway/immunohistochemistry‐an‐overview‐steps‐to‐better‐ihc‐staining

**Immunofluorescence:**

https://www.peprotech.com/de/protocols‐immunofluorescence‐general‐animal‐brain‐tissue

An open access article on immunofluorescence

Im K, Mareninov S, Diaz MFP, et al. An introduction to performing immunofluorescence staining. Methods Mol Biol. 2019;1897:299‐311.^227^
