# Supplementary material for: A companion to the preclinical common data elements and case report forms for neuropathology studies in epilepsy research. A report of the TASK3 WG2 Neuropathology Working Group of the ILAE/AES Joint Translational Task Force
Source: Epilepsia Open. 2022 Sep 22;10(Suppl 1):S112–35. doi: 10.1002/epi4.12638 (PMC12375993; doi:10.1002/epi4.12638)
Supplement: Supplementary file 1 — TABLE S1 [file EPI4-10-S112-s001.docx]

**Supplementary Table 1**

**Voting on Statements in CRFs**

*Voting-1 (4 working group members)*

“agreeing” “no opinion” “disagreeing” total

Statements votes votes votes votes

No (%) No (%) No (%) No

CRF module 1 44 150 (85) 21 (12) 5 (3) 176

CRF module 2 15 49 (15) 10 (17) 0 59

CRF module 3 14 51 (16) 5 (9) 0 56

CRF module 4 8 28 (9) 3 (10) 0 31

CRF module 5 5 16 (6) 4 (19) 1 (0.6) 21

Total 86 297 (92) 22 (7) 4 (1) 323

*Voting-2 (7 working group members)*

“agreeing” “no opinion” “disagreeing” total

Statements Votes Votes Votes Votes

No (%) No (%) No (%) No

CRF module 1 42 290 (99) 4 (1.3) 0 294

CRF module 2 17 119 (99) 1 (0.1) 0 120

CRF module 3 4 98 (100) 0 0 98

CRF module 4 9 63 (100) 0 0 63

CRF module 5 10 70 (100) 0 0 70

Total 90 640 (99) 5 (1) 0 645

Two voting cycles were performed. In the first voting, four group members participated, in the second one all seven members participated.

Statements voted as “no opinion” or “disagreeing” by one or more group members were discussed and either removed or changed. Two statements were removed and six statements added or detailed from previous ones as result of the discussion after the first voting.
