# Supplementary material for: A companion to the preclinical common data elements and case report forms for neuropathology studies in epilepsy research. A report of the TASK3 WG2 Neuropathology Working Group of the ILAE/AES Joint Translational Task Force
Source: Epilepsia Open. 2022 Sep 22;10(Suppl 1):S112–35. doi: 10.1002/epi4.12638 (PMC12375993; doi:10.1002/epi4.12638)
Supplement: Supplementary file 2 — Appendix S1 [file EPI4-10-S112-s002.zip › EPI4_12638_2 CRF Module Euthanasia and tissue preparation.docx]

Neuropathological Studies

Case Report Form

CRF module 2: Euthanasia and tissue preparation

Date that this CRF was filled out: Project name/Identifier:

Name of person filling out CRF: Animal ID:

| **CDE Name** | **Data Collected** |
| --- | --- |
| **Method of euthanasia** | |
| Method of euthanasia chemical method | ☐ CO_2_ followed by decapitation  ☐ Anesthesia ☐ Ketamine/Xylazine, ☐ Phenobarbital  Unknown  Other |
| If other chemical method used, please specify |  |
| If anesthesia used, which type | ☐ Isoflurane ☐ Sevoflurane Unknown  Other |
| If other anesthesia used, please specify |  |
| Method of euthanasia physical method | ☐ Decapitation Unknown ☐ Other |
| If other physical method used, please specify |  |
| Date of euthanasia (DD/MM/YYYY) |  |
| **Tissue selection & preparation** | |
| Indicate tissue selection | ☐ Whole brain  ☐ Hippocampus ☐ Amygdala ☐ Cortex ☐ Midbrain  ☐ Cerebellum  Unknown  ☐ Other |
| If other tissue selection, please specify |  |
| State method of tissue preparation | ☐ Fresh  Rapid freezing  Fixation |
| State method of rapid freezing | ☐ Dry ice ☐ Liquid nitrogen  ☐ Immersion into isopentane, -80 °C |
| State fixative used | Paraformaldehyde  ☐ Glutaraldehyde ☐ Formalin Unknown ☐ Other |
| If other please specify |  |
| If paraformaldehyde used, specify concentration (%) | Commonly used: 4%  ……% |
| If glutaraldehyde, specify concentration (%) | Minimum: 0.1 %, maximum: 2%  ……..% |
| If paraformaldehyde and glutaraldehyde, specify concentrations (%) | Commonly used: paraformaldehyde 4%, glutaraldehyde 0.2 %  Paraformaldehyde: ….%, glutaraldehyde: % |
| Indicate mode of fixation | ☐ Immersion fixation  ☐ Whole animal fixative vascular perfusion |
| If immersion fixation used, please specify duration (hrs, days) | Minimum: 2 hrs, maximum: 14 days  ………… |
| If immersion fixation used, please specify temperature (^o^C) | Minimum: 4°C, Maximum: room temperature (25 C°)  …………. |
| If whole animal fixative vascular perfusion, specify gravity (mm Hg) | Typically: 80 to 130 mm Hg |
| If whole animal fixative vascular perfusion, specify pump model & supplier |  |
| Post-perfusion | ☐ Overnight *in situ* ☐ Immersion in same fixative  ☐ Cryoprotection |
| Post-perfusion, state solution |  |
| Post-perfusion, state duration (hrs) | Typically, 1 to 48 hrs |
| Post-perfusion, state date of tissue fixation (DD/MM/YYYY) |  |
| **Storage of brain tissue** | |
| Storage of brain tissue if fresh | ☐ Freezer (-80°C) ☐ Freezer (-20°C) Unknown Other |
| If other please specify |  |
| Storage of brain tissue if fixated dry | ☐ Freezer (-80°C) ☐ Freezer (-20°C) ☐ Refrigerator (4°C)  Unknown Other |
| If other type of storage, please specify |  |
| Storage of brain tissue if in solution | ☐ Refrigerator (4°C) PBS/ 0.01 % **sodium azide**  ☐ Freezer (-20°C) ☐ DMSO glycerol ☐ Glycerol Unknown  Other |
| If other please specify |  |
| Duration of storage, please specify (days) | Typically, 1 day to several months  ……… |
| Sectioning type | ☐ Cryostat ☐ Vibratome ☐ Microtome |
| Specify company/model of instrument |  |
| Specify temperature of sectioning (^o^C) | Typically -5 to 8 C° (cryostate), 20 to 25 C° (Vibratome, microtome)  …………. |
| Specify thickness of sections in µm | Typically 7 to 30 µm  ……. |
| Section processing during staining | ☐ Free floating ☐ Frozen section mounted on glass slides  ☐ Paraffin Unknown ☐ Other |
| If other please specify |  |
| **Data archiving/repository**  **for tissue/sections** | |
| Data archiving/repository  please state box number. Upload file protocol |  |

I**nstructions**

Please check mark with a cross where applicable. If none of the predetermined options is appropriate use the default space to specify your answer.

The form is to be filled in for one individual animal.
