# Supplementary material for: A companion to the preclinical common data elements and case report forms for neuropathology studies in epilepsy research. A report of the TASK3 WG2 Neuropathology Working Group of the ILAE/AES Joint Translational Task Force
Source: Epilepsia Open. 2022 Sep 22;10(Suppl 1):S112–35. doi: 10.1002/epi4.12638 (PMC12375993; doi:10.1002/epi4.12638)
Supplement: Supplementary file 2 — Appendix S1 [file EPI4-10-S112-s002.zip › EPI4_12638_3 CRF Module Neurodegeneration-rev.docx]

Neuropathological Studies

Case Report Form

CRF module 3: Neurodegeneration

Pathology associated with neurons and neuronal pathways

Date that this CRF was filled out: Project name/Identifier:

Name of person filling out CRF: Animal ID:

| **CDE Name** | **Data Collected** |
| --- | --- |

| **Stains to identify cell death in general** | | | |
| --- | --- | --- | --- |
| Staining | ☐ Hoechst ☐ DAPI ☐ Fluoro Jade B. ☐ Fluoro Jade C  ☐ Other | | |
| If other, please specify |  | | |
| **Markers for investigating changes in functional components of neurons** | | | |
| **Stains for perikarya** | ☐ Nissl ☐ H&E ☐ DAPI ☐ NeuN ☐ Other | | |
| If other, please specify |  | | |
| **Specific neuronal markers** | | | |
| **Dendrites and axons** | ☐ MAP-2 ☐ Neurofilaments ☐ Other | | |
| If other, please specify |  | | |
| Specify method used | ☐ Direct immunohistochemistry (IH) ☐ Indirect IH  ☐ Immunofluorescence (IF) ☐ *In situ*-hybridization (ISH)  ☐ Immunoblotting (IB) | | |
| **Subtypes of neurons** | | | |
| **Pyramidal neurons** | ☐ VGLUT1 ☐ VGLUT2 ☐ CaMKII M | | |
| Specify method used | ☐ Direct IH ☐ Indirect IH ☐ IF ☐ ISH ☐ IB | | |
| **GABA-ergic interneurons** | ☐ GAD1 ☐ GAD2 ☐ VGAT | | |
| **Subtypes of GABA neurons** | ☐ Parvalbumin ☐ Somatostatin ☐ NPY ☐ CCK ☐ VIP ☐ Other | | |
| If other, please specify |  | | |
| Specify method used | ☐ Direct IH ☐ Indirect IH ☐ IF ☐ ISH ☐ IB | | |
| **Activated neurons** | | | |
| Marker | ☐ *cfos:* ISH ☐ FosB ☐ *Arc*  ISH | | |
| If FOSB, specify method | ☐ Direct IH ☐ Indirect IH ☐ IF | | |
|  | | | |
| **Techniques to characterize cell death type** | | | |
| **Cell death type to be investigated** |  | | |
| **Mechanism of accidental cell death:** |  | | |
| **Necrosis** | ☐ Electron microscopy  ☐ Other | | |
| If other, please specify |  | | |
| **Mechanisms of regulated cell death (RCD):** |  | | |
| **Necroptosis** | EM combined with ☐ Immunoblotting (IB) ☐ IHC  ☐ RIPK1 ☐ RIPK3 ☐ p-MLKL  ☐ Other | | |
| If other, please specify |  | | |
| **MPT-driven necrosis (**EM combined with inhibition by cyclophilin D inhibitors) | ☐ Cyclosporin A ☐ Sanglifehrin A ☐ JW47  ☐ Other | | |
| If other, please specify |  | | |
| **Ferroptosis and Lipid peroxidation**  EM combined with: | ☐ Pearl staining  ☐ Iron tissue content  ☐ GPX4 IHC (decrease)  ☐ PTGS2 RT-PCR  levels for ☐ GSH and ☐ MDA  combined with inhibition by ferroptosis inhibitors:  ☐ ferrostatin-1 ☐ other | | |
| If other, please specify |  | | |
| **Intrinsic apoptosis** | ☐ Cytochrome c leakage by IH/immune EM  ☐ CASP3 ☐ CASP9 ☐ CASP7 ☐ Other | | |
| If other, please specify |  | | |
| Specify method used | ☐ Direct IH ☐ Indirect IH ☐ IF | | |
| **Extrinsic apoptosis** | ☐ EM ☐ CASP8 IHC ☐ CASP8 IB  ☐ Inhibited by CASP8 inhibitors (z-IETD-fmk)  ☐ Other | | |
| If other, please specify |  | | |
| **Autophagy** | ☐ Labeling of autophagic vacuoles for LC3  Method used: ☐ EM and IHC ☐ Immune-EM  ☐ Other | | |
| If other, please specify |  | | |
| **Taupathy**  (Marker) | ☐ AT8 Hyperphosphorylated tau (AT8) ☐ AT270 (pThr181)  ☐ AT100 (pSer212/pSer214) ☐AT180 (pThr231) ☐PHF-6 (pThr231) ☐1H6L6 (pThr231) ☐ anti-tau (pSer404)  ☐ Amyloid-beta (βA4) | | |
| Specify method used | ☐ Direct IH ☐ Indirect IH ☐ IF ☐ ISH ☐ IB | | |
| **Proliferating neurons in the hilus of the dentate gyrus** | | |  |
| Unspecific: All states | ☐ labelling of neurons after BrdU injection | |  |
| Early state | ☐ Ki67 ☐ Nestin | |  |
| Specify method used | ☐ Direct IH ☐ Indirect IH ☐ IF ☐ ISH | |  |
| Intermediate and late state | ☐ DCX ☐ Calretinin ☐ NeuN | |  |
| Specify method used | ☐ Direct IH ☐ Indirect IH ☐ IF ☐ ISH | |  |
| **Quantification of data** | | |  |
| Mode of quantification | | ☐ Descriptive (data not quantified)  ☐ Semi-quantitative  ☐ Qualitative  ☐ Quantitative  Stereology  ☐ Automatic reconstruction of 3D cell morphology  ☐ Densitometry  ☐ Other procedures |  |
| State marker quantified | |  |  |
| State method used for quantification | | ☐ Immunohistochemistry (IHC)  ☐ In situ hybridization (ISH) ☐ Immunoblot (IB)  ☐ RT-PCR ☐ Other |  |
| If other method used, please specify | |  |  |
| State quantitative data (e.g. counts per mm^3^) | |  |  |
| **Archive** | | |  |
| Data archiving/repository  please state box number. Upload file protocol | |  |  |

**Instructions**

Please check mark with a cross where applicable. If none of the predetermined options is appropriate, use the default space to specify your answer.

The form is to be filled in for one individual animal.
