# Supplementary material for: A companion to the preclinical common data elements and case report forms for neuropathology studies in epilepsy research. A report of the TASK3 WG2 Neuropathology Working Group of the ILAE/AES Joint Translational Task Force
Source: Epilepsia Open. 2022 Sep 22;10(Suppl 1):S112–35. doi: 10.1002/epi4.12638 (PMC12375993; doi:10.1002/epi4.12638)
Supplement: Supplementary file 2 — Appendix S1 [file EPI4-10-S112-s002.zip › EPI4_12638_4 CRF Module Astrocyte pathology.docx]

Neuropathological Studies

Case Report Form

CRF module 4: Astrocyte pathology

Date that this CRF was filled out: Project name/Identifier:

Name of person filling out CRF: Animal ID:

| **CDE Name** | **Data Collected** |
| --- | --- |

| **Stains to identify cell death** | |
| --- | --- |
| Type of stains to identify cell death | ☐ Hoechst ☐ DAPI ☐ Fluoro Jade B. ☐ Fluoro Jade C  ☐ Other |
| If other stains used, please specify |  |
| **Stains to identify proliferation** | |
| Type of stains to identify proliferation | ☐ Ki67 (IHC) ☐ PCNA (IHC) ☐ BrdU ☐ EdU  ☐ Other |
| If other stains used, please specify |  |
| **Characterize affected astrocytes** | |
| Type of affected astrocytes  Immunohistochemistry (IH) | ☐ GFAP ☐ S100b ☐ ALDH1L1 ☐ GLAST  ☐ GLT-1 ☐ Vimentin ☐ AQP4 ☐ Cx43  ☐ K_ir_4.1 ☐ Other |
| If other affected astrocytes, please specify |  |
| **Techniques to characterize astrocyte death type** | |
| **Necrosis**  Marker (IH) | ☐ Pl ☐ Other |
| If other necrosis, please specify |  |
| **Necroptosis**:  Marker (IH) | ☐ RIPK3 ☐ MLKL ☐ p-MLKL ☐ cFLIP  ☐ HSP90a ☐ TRAP1 ☐ Other |
| If other, please specify |  |
| **Apoptosis**  Marker (IH) | ☐ CASP3 ☐ CASP8 ☐ TUNNEL ☐ Other |
| If other, please specify |  |
| **Autophagy**  Immunohistochemistry for autophagosome marker | ☐ LC3B ☐ LAMP2 ☐ BECN1  ☐ Other |
| If other, please specify |  |
| **Other cell death forms** |  |
| Type of method used | ☐ EM ☐ IHC/immune-EM ☐ IB ☐ RT-PCR ☐ ISH |
| **Quantification of data** | |
| **Mode of quantification** | ☐ Descriptive (data not quantified)  ☐ Semi-quantitative  ☐ Qualitative  ☐ Quantitative  Stereology  ☐ Automatic reconstruction of 3D cell morphology  ☐ Densitometry  ☐ Other procedures |
| State marker quantified |  |
| State method used for quantification | ☐ Immunohistochemistry (IHC)  ☐ In situ hybridization (ISH) ☐ Immunoblot (IB)  ☐ RT-PCR ☐ Other |
| If other method used, please specify |  |
| State quantitative data (e.g. counts per mm^3^) |  |
| Data archiving/repository  for tissue/sections, please state box number. Upload file protocol |  |

**Instructions**

Please check mark with a cross where applicable. If none of the predetermined options is appropriate use the default space to specify your answer.

The form is to be filled in for one individual animal.
