# Supplementary material for: A companion to the preclinical common data elements and case report forms for neuropathology studies in epilepsy research. A report of the TASK3 WG2 Neuropathology Working Group of the ILAE/AES Joint Translational Task Force
Source: Epilepsia Open. 2022 Sep 22;10(Suppl 1):S112–35. doi: 10.1002/epi4.12638 (PMC12375993; doi:10.1002/epi4.12638)
Supplement: Supplementary file 2 — Appendix S1 [file EPI4-10-S112-s002.zip › EPI4_12638_5 CRF Module Immune responses.docx]

Case Report Form

CRF module 5: Epilepsy-induced immune response*s*

Date that this CRF was filled out: Project name/Identifier:

Name of person filling out CRF: Animal ID:

| **CDE Name** | **Data Collected** |
| --- | --- |

| **A.** **Lectin-horseradish peroxidase stain for microglia** | |
| --- | --- |
| Stains to identify cell death: Lectin-horseradish peroxidase | ☐ Yes ☐No |
| **B. Immunohistochemistry (IH)** | |
| **Microglia** | ☐ Iba1 ☐ CD11b ☐ Tmem119  ☐ Cx3cr1 |
| Please indicate method used | ☐ Direct IH ☐ Indirect IH ☐ Immunofluorescence (IF) |
| **Monocyte/Macrophage** | ☐ CD68 ☐ CD163 |
| Please indicate method used | ☐ Direct IH ☐ Indirect IH ☐ IF |
| **B-lymphocyte (**CD20) | ☐ Yes ☐ No |
| Please indicate method used | ☐ Direct IH ☐ Indirect IH ☐ IF |
| **T-lymphocyte** | ☐ CD3 ☐ CD4 ☐ CD8 |
| Please indicate method used | ☐ Direct IH ☐ Indirect IH ☐ IF |
| **Dendritic cells** | ☐ CD11b ☐ CD83 ☐ CD209 |
| Please indicate method used | ☐ Direct IH ☐ Indirect IH ☐ IF |
| **Natural killer cell** | ☐ NKp46 |
| Please indicate method used | ☐ Direct IH ☐ Indirect IH ☐ IF |
| **C. Double labeling** | |
| Cells investigated | ☐ Neuronal ☐ Glial ☐ Endothelial |
| Please indicate markers used |  |
| **D. Immunohistochemistry or *in situ*-hybridization** | |
| Marker investigated | ☐ Cytokines ☐ Chemokines ☐ Chemokines receptors  ☐ Implement factors |
| If Cytokine, state which and method used | ☐ Direct IH ☐ Indirect IH ☐ IF ☐ In situ-hybridization (ISH) |
| If Chemokine, state which and method used | ☐ Direct IH ☐ Indirect IH ☐ IF ☐ In situ-hybridization (ISH) |
| If Chemokine receptors, state which and method used | ☐ Direct IH ☐ Indirect IH ☐ IF ☐ In situ-hybridization (ISH) |
| If Implement factors, state which and method used | ☐ Direct IH ☐ Indirect IH ☐ IF ☐ In situ-hybridization (ISH) |
| **E. In situ-hybridization** | |
| Type of RNA investigated | ☐ miRNA, ☐ cRNA, ☐ lcRNA,  ☐ lncRNA |
| **Quantification of data** | |
| Mode of quantification | ☐ Descriptive (data not quantified)  ☐ Semi-quantitative  ☐ Qualitative  ☐ Quantitative  Stereology  ☐ Automatic reconstruction of 3D cell morphology  ☐ Densitometry  ☐ Other procedures |
| State marker quantified |  |
| State method used for quantification | ☐ Immunohistochemistry (IHC)  ☐ In situ hybridization (ISH) ☐ Immunoblot (IB)  ☐ RT-PCR ☐ Other |
| If other method used, please specify |  |
| State quantitative data (e.g. counts per mm^3^) |  |
| **Archive** |  |
| Data archiving/repository  for tissue/sections, please state box number. Upload file protocol |  |

**Instructions**

Please check mark with a cross where applicable. If none of the predetermined options is appropriate use the default space to specify your answer.

The form is to be filled in for one individual animal.
