# Supplementary material for: A companion to the preclinical common data elements and case report forms for neuropathology studies in epilepsy research. A report of the TASK3 WG2 Neuropathology Working Group of the ILAE/AES Joint Translational Task Force
Source: Epilepsia Open. 2022 Sep 22;10(Suppl 1):S112–35. doi: 10.1002/epi4.12638 (PMC12375993; doi:10.1002/epi4.12638)
Supplement: Supplementary file 2 — Appendix S1 [file EPI4-10-S112-s002.zip › EPI4_12638_6 CRF Module Oligodendrocytes myelin BBB.docx]

Case Report Form

CRF module 6: Oligodendrocytes, myelin and BBB

Date that this CRF was filled out: Project name/Identifier:

Name of person filling out CRF: Animal ID:

| **CDE Name** | **Data Collected** |
| --- | --- |

| **Oligodendrocytes and myelin** | |
| --- | --- |
| **Oligodendrocyte and myelin markers** (immunohistochemistry IH) | ☐ CNPT ☐ CNP ☐ CC1 ☐ GSTpi ☐ PLP  ☐ MAG ☐ MOG ☐ MBP ☐ Luxol fast blue ☐ Other |
| If other, please specify |  |
| **Blood-brain barrier (BBB)** | |
| **Marker for plasma extravasation** | ☐ Evan’s blue method ☐ Fluorescein-albumin ☐ HRP  ☐ Other |
| If other, please specify |  |
| **Endothelial cell marker** (immunohistochemistry) | ☐ CD31 ☐ Tie2 ☐ RECA-1 ☐ UEA-I lectin |
| **Pericyte marker** (immunohistochemistry) | ☐ PDGFRb ☐ NG2 |
| **Data archiving/repository**  please state box number. Upload file protocol |  |

**Instructions**

Please check mark with a cross where applicable. If none of the predetermined options is appropriate use the default space to specify your answer.

The form is to be filled in for one individual animal.
